# Supplementary material for: Causal Relationship between Aging and Anorexia Nervosa: A White-Matter-Microstructure-Mediated Mendelian Randomization Analysis
Source: Biomedicines. 2024 Aug 16;12(8):1874. doi: 10.3390/biomedicines12081874 (PMC11351342; doi:10.3390/biomedicines12081874)
Supplement: Supplementary file 1 [file biomedicines-12-01874-s001.zip › Supplementary Figure/Figure S4/Singe SNP plot of MR analysis for TL to ubm-b-1578.pdf]

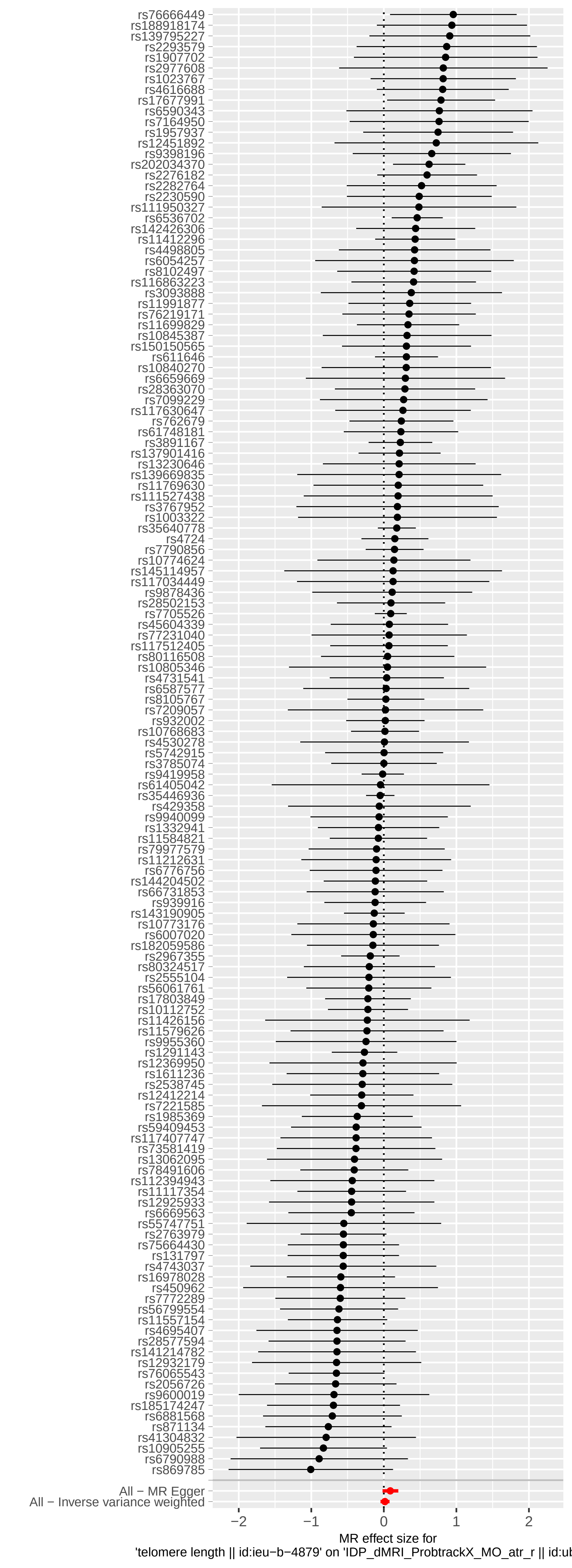
rs76666449
rs188918174
rs139795227
rs2293579
rs1907702
rs2977608
rs1023767
rs4616688
rs17677991
rs6590343
rs7164950
rs1957937
rs12451892
rs9398196
rs202034370
rs2276182
rs2282764
rs2230590
rs111950327
rs6536702
rs142426306
rs11412296
rs4498805
rs6054257
rs8102497
rs116863223
rs3093888
rs11991877
rs76219171
rs11699829
rs10845387
rs150150565
rs611646
rs10840270
rs6659669
rs28363070
rs7099229
rs117630647
rs762679
rs61748181
rs3891167
rs137901416
rs13230646
rs139669835
rs11769630
rs111527438
rs3767952
rs1003322
rs35640778
rs4724
rs7790856
rs10774624
rs145114957
rs117034449
rs9878436
rs28502153
rs7705526
rs45604339
rs77231040
rs117512405
rs80116508
rs10805346
rs4731541
rs6587577
rs8105767
rs7209057
rs932002
rs10768683
rs4530278
rs5742915
rs3785074
rs9419958
rs61405042
rs35446936
rs429358
rs9940099
rs1332941
rs11584821
rs79977579
rs11212631
rs6776756
rs144204502
rs66731853
rs939916
rs143190905
rs10773176
rs6007020
rs182059586
rs2967355
rs80324517
rs2555104
rs56061761
rs17803849
rs10112752
rs11426156
rs11579626
rs9955360
rs1291143
rs12369950
rs1611236
rs2538745
rs12412214
rs7221585
rs1985369
rs59409453
rs117407747
rs73581419
rs13062095
rs78491606
rs112394943
rs11117354
rs12925933
rs6669563
rs55747751
rs2763979
rs75664430
rs131797
rs4743037
rs16978028
rs450962
rs7772289
rs56799554
rs11557154
rs4695407
rs28577594
rs141214782
rs12932179
rs76065543
rs2056726
rs9600019
rs185174247
rs6881568
rs871134
rs41304832
rs10905255
rs6790988
rs869785
All − MR Egger
All − Inverse variance weighted
−2
−1
0
1
2
MR effect size for
'telomere length || id:ieu−b−4879' on 'IDP_dMRI_ProbtrackX_MO_atr_r || id:ub
